# Cell-Free Therapies: Novel Approaches for COVID-19

**DOI:** 10.3389/fimmu.2020.583017

**Published:** 2020-09-18

**Authors:** Tatiana Maron-Gutierrez, Patricia R. M. Rocco

**Affiliations:** ^1^Laboratory of Immunopharmacology, Oswaldo Cruz Institute, Oswaldo Cruz Foundation, Fiocruz, Rio de Janeiro, Brazil; ^2^National Institute of Science and Technology for Neuroimmunomodulation, Rio de Janeiro, Brazil; ^3^Laboratory of Pulmonary Investigation, Carlos Chagas Filho Institute of Biophysics, Federal University of Rio de Janeiro, Rio de Janeiro, Brazil; ^4^National Institute of Science and Technology for Regenerative Medicine, Rio de Janeiro, Brazil; ^5^COVID-19 Virus Network, Ministry of Science and Technology, and Innovation, Rio de Janeiro, Brazil

**Keywords:** COVID-19, SARS-CoV-2, cell-free therapies, extracellular vesicle (EV), MSC secretome, mitochondria

## Introduction

First described in late 2019, coronavirus disease (COVID)-19, caused by the severe acute respiratory syndrome coronavirus 2 (SARS-CoV-2), rapidly escalated into a global pandemic with a high case fatality rate. COVID-19 patients with acute respiratory failure exhibit some distinctive pathological characteristics, which to some extent resemble the acute respiratory distress syndrome (ARDS), SARS, and Middle East respiratory syndrome (MERS); these include hypoxemia, diffuse alveolar damage with cellular exudates, extensive pulmonary inflammation, lung edema, and hyaline membrane formation (1). In addition to respiratory failure, these patients present with a dysfunctional systemic host response that affects multiple organs, including the central nervous, cardiovascular, renal, and gastrointestinal systems (2–4), as well as a wide range of coagulation disturbances, such as thrombocytopenia, sustained systemic clotting activation, massive thrombin and fibrin formation, and disseminated intravascular coagulation (5).

## Current Available Treatments Against COVID-19

Few therapies are effective for COVID-19 patients, and to date there are still no vaccines available. There are vaccine candidates in development and ongoing clinical trials, but they are expected to be available, at best, in early 2021 (6). Thus, novel effective and safe therapies are urgently required to treat COVID-19 patients (7). In this context, several clinical trials have begun pursuing new therapies as well as repurposing existing ones, largely through drug repositioning, including of antiviral, antimalarial, and anti-inflammatory agents. These therapies aim to target entry of the virus into host cells, multiplication of the viral genetic material, and/or the immune response and inflammatory process (6). Although some therapies shows promising results, they raise some concerns, such as limited cohort sizes, non-randomized trials, lack of considerations for gender, comorbidities, concurrent treatments, and route of drug delivery, among others (6). Early in the COVID-19 pandemic, corticosteroids were not recommended because of the adverse effects previously observed in influenza, SARS-CoV, and MERS-CoV infections (8). Nevertheless, a recent controlled, open-label trial of dexamethasone for up to 10 days resulted in lower 28-day mortality in mechanically ventilated patients (9).

**Figure 1:**
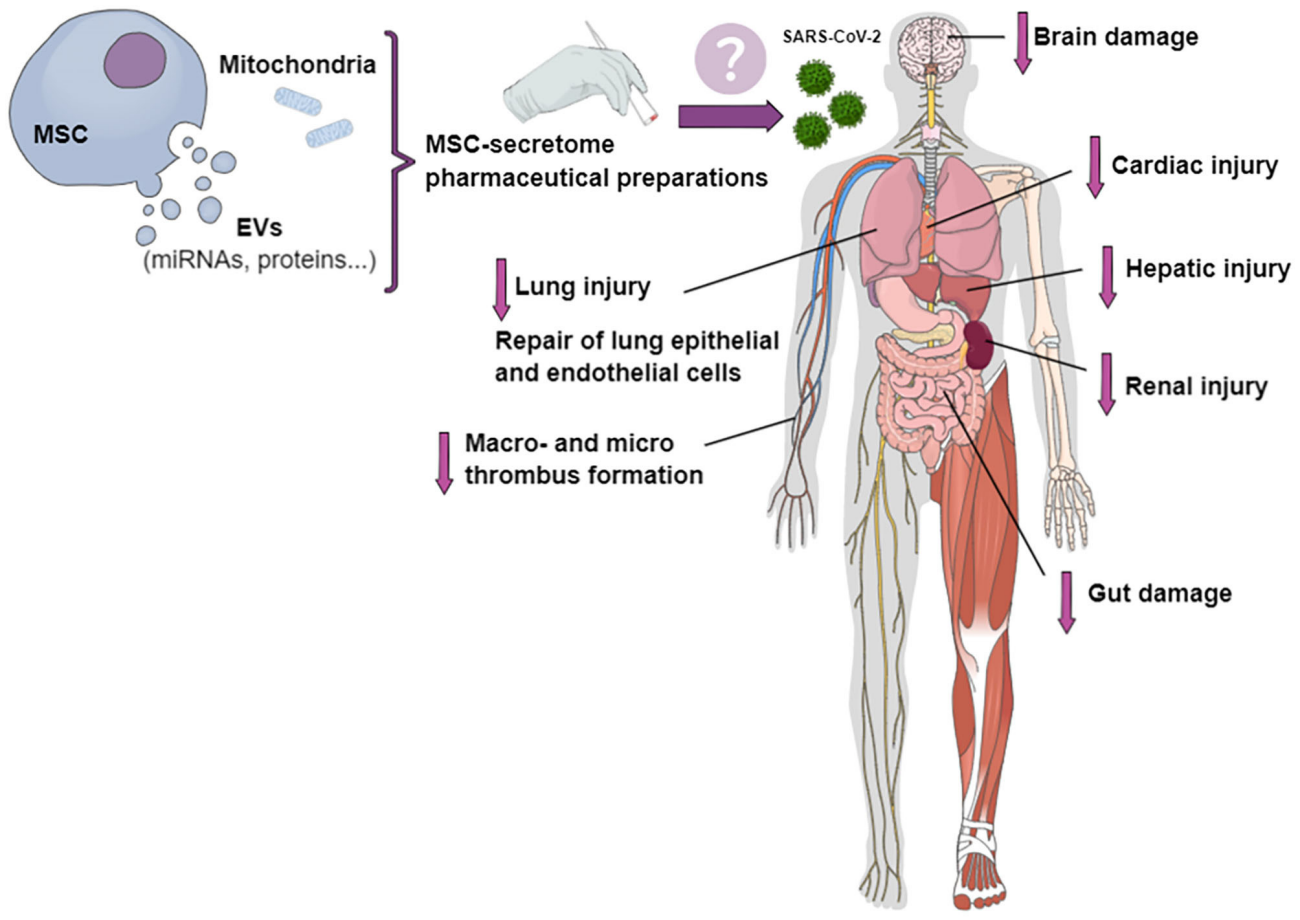
Potential effects of pharmaceutical preparations of MSC secretome on different organs involved in COVID-19. The MSC secretome, in the form of conditioned medium containing extracellular vesicles (EVs) and mitochondria, could be transformed into a stable product for the treatment of patients with COVID-19. Secretome-based therapies might mitigate cardiac, kidney, liver, nervous system, and lung injury; decrease macro- and micro-thrombus formation and endothelial inflammation; and repair lung epithelial and endothelial cells.

Mesenchymal stromal cell (MSC)-based treatment has been proposed as a suitable therapeutic approach for COVID-19 (10). As MSCs have the potential to interact directly with immune cells, their transplantation may improve outcomes in COVID-19 patients through modulation of the immune response, mitigation of the inflammatory cascade, and promotion of tissue repair and regeneration (11, 12). In addition, CD147, the second entry receptor for SARS-CoV-2, can be expressed by tissue-specific stem cells (13). Together with the loss of airway epithelial cells by viral infection and replication, the additional loss of regenerating stem cells may be responsible for diminished cellular and lung regeneration (13). Cell-based therapies have been quite extensively studied for potential applicability in COVID-19, especially given the short time since the onset of the pandemic (14, 15); however, due to the risk of macro- and microthrombosis, cell-free therapies may be more appealing. Cell-free therapies might decrease injury to different organs, such as lung, heart, kidney, liver, and brain, as well as reduce thrombus formation and endothelial inflammation (Figure 1).

## Cell-Free Therapies for COVID-19

Cell-free therapies, such as the MSC secretome (obtained as conditioned medium) and extracellular vesicles (EVs) from MSCs, have been studied in ARDS (16) and multiple organ dysfunction syndrome (MODS) (17, 18) for their anti-inflammatory and anti-fibrogenic effects, as well as their epithelial and endothelial regenerative properties. However, many researchers and international societies, including the International Society for Extracellular Vesicles (ISEV) and the International Society for Cellular and Gene Therapies (ISCT), have expressed concern regarding the use of EVs—whether derived from MSCs or from other cell sources—in the treatment of COVID-19 (19). Clinical trials are encouraged; however, the use of EVs for any purpose in COVID-19 is not endorsed by ISEV and ISCT until proper regulation of manufacturing, quality control protocols, and clinical trial design are in place, in order to avoid the stem-cell industry trying to sell unregulated MSC treatments (19). In this context, the implementation of computer-controlled bioreactors (20) and the development of standard operating procedures (SOPs) for obtaining a Good Manufacturing Practice (GMP)-grade MSC secretome and its components are necessary for clinical applications (21). These must be reproducible, scalable, and well-controlled to limit heterogeneity and enhance predictability in the composition and function of secretome-derived products (22). Further studies are still needed to better understand the best route of cell-free therapy delivery, dose, and timing of administration (23). Moreover, important factors should be taken into consideration such as the culture medium, cell and tissue source, donor variability, and culture conditions (cell priming with hypoxia, biochemical or mechanical stimuli, three-dimensional spheroid culture, among others), as well as the timing and method of MSC-secretome harvesting (20, 22). In short, cell-free therapies could be a more suitable treatment for COVID-19 than MSCs, but additional investigations are required (2).

## Therapeutic Strategies Involving the MSC Secretome in COVID-19

The MSC secretome is a complex mixture of soluble components (growth factors and cytokines), a vesicular portion that comprises EVs, and cell organelles (e.g., mitochondria) (24–26). Considering that SARS-CoV-2 infection is being associated with an increased inflammatory process (27), we believe that MSC secretome products might help reverse COVID-19–related immune dysregulation, due to their anti-inflammatory, immunomodulatory, and regenerative effects (14). The MSC secretome has properties similar to those of its parent MSCs (28). Moreover, the secretome is generally considered safer than parent cells, since it (1) lacks the potential for endogenous tumor formation, as it cannot self-replicate; (2) can be classified as non-immunogenic, due to the limited number of antigenic components; and (3) may lead to less formation of emboli when injected intravenously (14, 29). As recently reported elsewhere, the MSC secretome (in the form of conditioned medium) can be stored more easily than MSCs (14), which is an important consideration given the lack of adequate facilities in developing countries. Transforming MSC-secretome components into a freeze-dried, stable powder product which can be reconstituted for intravenous injection or inhalation might be a suitable approach for the treatment of patients with COVID-19 (Figure 1) (17).

## Mitochondria for Restoring Bioenergetics and Mitigating Inflammation

Mitochondria are intracellular organelles that play a vital role in cellular homeostasis and enable stress adaptation (30). Most cellular energy generation takes place in the mitochondria (31), and excessive mitochondrial dysfunction leading to defects in energy flow leads to unsustainable maintenance of life and adaptation to stress (30). One of the main mechanisms associated with the pathophysiology of sepsis is mitochondrial dysfunction (32, 33). In 2012, the first evidence that MSCs restore alveolar bioenergetics through Cx43-dependent alveolar attachment and mitochondrial transfer was observed in experimental ARDS (25). In 2015, Phinney et al. observed mitochondrial transfer from MSCs to macrophages in response to oxidative stress (34). Recently, Court et al. investigated the effect of MSC-mediated transfer of mitochondria on lymphoid cells. They observed mitochondria-labeled MSCs mainly in CD4+T cells, paving the way for exploration of organelle-based therapies in immune diseases (26). Interestingly, MSCs are not the only cell type able to transfer mitochondria. Lipopolysaccharide (LPS)-stimulated monocytes release free and microvesicle-associated mitochondria as part of their secretome (35). These studies demonstrate the complexity of cell-to-cell communication by identifying mitochondria as a source for target cells to restore their bioenergetics, enable immunomodulatory effects, and suppress inflammation. Clinical trials failed to show efficacy of immunomodulatory therapies in sepsis (36, 37). Since bacterial sepsis shares some similarities with COVID-19, we may consider a new route for therapeutic intervention focused on mitochondrial cell transfer. Another option is therapy with engineered EVs containing mitochondria.

## Rationale for Using Extracellular Vesicles in COVID-19

The immunomodulatory and regenerative potential of MSCs may be independent of direct cellular cross-talk (21, 38). MSCs act through a paracrine mechanism based primarily on EVs, which interact with neighboring target cells or can reach distant organs (39). Distinctions between the subtypes of EVs were previously based on subcellular origin, with exosomes being of endosomal origin and microvesicles derived from the cell membrane. However, given the historically contradictory definitions and inaccurate expectations of biogenesis associated with these terms, in 2018, ISEV recommended the use of new terms for EV subtypes that refer to their physical characteristics, such as size (small and medium/large EVs) or density; their biochemical composition (CD63+/CD81+- EVs, annexin A5-stained EVs, etc.); or their cell of origin (MSC EVs, podocyte EVs, etc.) (40). EV biochemistry varies according to composition and cell source (41). EVs can carry membrane and cytosolic proteins, transcription factors, DNA, coding and non-coding RNAs and various signal transduction molecules (5, 21, 38, 42), acting on both physiological and pathological events, e.g., modulating the inflammatory response (2, 14). EVs also carry different cytokines and growth factors, such as interleukin (IL)-6 and IL-10, transforming growth factor (TGF)-β, and hepatocyte growth factor (HGF) (43). In addition, EVs contain matrix-remodeling enzymes, such as matrix metalloproteinases (MMPs), heparanases, hyaluronidases, and tissue inhibitors of metalloproteinases (TIMPs); EV-mediated proteolytic activities have also been described, which might modulate the remodeling process and contribute to tissue repair (39). In this context, our group demonstrated that MSCs increased MMP-8 expression and decreased TIMP-1 expression in an experimental model of ARDS, suggesting an effect on the extracellular matrix (44). Rather than suppressing immune responses, EVs appear to act as true modulators, inducing regulatory responses and tolerance in order to restore homeostasis (45).

Administration of EVs has proven safe and effective in preclinical studies of lung injury and sepsis models (16, 18, 19, 46–48). In preclinical studies, EV therapy ameliorated acute lung injury (49, 50) and was equally or even more effective than MSCs in mitigating lung inflammation and pathological damage (41, 51). EVs have also been shown to attenuate *E. coli* and influenza infections (47, 48, 52), including a mixed swine (H3N2, H1N1) and avian (H9N5, H7N2) influenza-induced lung injury model (48). The beneficial effects of EVs have further been observed in an ischemic stroke model (53, 54). Since COVID-19 may be associated with damage to other organs in addition to the lungs and has been associated with ischemic stroke, EVs could be a particularly promising therapy in this context (19). However, only one prospective study has evaluated the effects of EVs (specifically, exosomes from bone marrow-derived MSCs) in COVID-19 patients (55). Even though the inflammatory response was reduced significantly, and no adverse events were observed, this study encountered limitations regarding EV characterization and biological properties, the actual dose of EV administered, and how the injection of EVs was monitored. At the time of writing, there are three ongoing clinical trials of MSC-derived EVs for COVID-19 treatment, to be administered intravenously (ChiCTR2000030484) or by inhalation (NCT04276987, ChiCTR2000030261); however, recruitment has not yet begun.

It is important to consider both the source of MSCs from which EVs are derived, which can be obtained from different tissues and donors, and their preparation. Depending on these factors, MSCs and their EVs can have different therapeutic properties. Compared to bone marrow-derived MSCs, for instance, adipose tissue-derived MSCs express more tissue factor (an important initiator of coagulation in sepsis) and reduce hemocompatibility, which has been shown to vary according to donor and culture handling conditions (56, 57). Therefore, EVs obtained from adipose tissue-derived MSCs may have greater thrombogenic activity than those from bone marrow-derived MSCs (58, 59), and thus should not be considered for use in COVID-19 patients (5, 19). Furthermore, the therapeutic effects of EVs are known to vary according to their preparation method, even when obtained from the same MSC source (53). Moreover, differences in donor parameters, including age, have been associated with significant variations in cytokine content, thus resulting in different effects on injury mitigation (60, 61).

In addition to their natural cargo, EVs can be loaded with biochemical compounds or genetically engineered to target infected cells, thus providing additional perspective for COVID-19 treatment beyond MSC-derived EVs, including EV-based drug delivery, inhibition of EV biogenesis and uptake, and EV-based vaccines (41). The latter might be a particularly promising cell-free approach to COVID-19 treatment. Exosome vaccines may contain membrane-anchored ectodomains of SARS-CoV-2 components on their surface, facilitating cross-linking of the B-cell receptor (2). Exosome-based vaccines containing the spike (S) proteins of SARS-CoV-2, one of the structural proteins that mediate viral entry into the host cells (62), could induce high levels of neutralizing antibodies (63). In addition, the cargo of EV-based vaccines can be modified to include proteins and miRNAs to help modulate the immune response (62). However, further research is needed to assess the safety and clinical pharmacology of EV-based therapies in order to provide guidance for manufacturing, storage, dosing, and administration (19) before these potential treatments can be made more accessible worldwide (14, 17).

## Conclusion

In the specific setting of COVID-19, administration of MSC-EVs may have several advantages over MSCs: (i) there is no risk of emboli formation in the injured microcirculation; (ii) no risk of mutagenicity or oncogenicity is observed; (iii) nebulized delivery can be used (despite several controversies regarding this route of administration); and (iv) tolerance of longer storage periods allow for later therapeutic use, reducing the stringency of storage and transportation requirements. In short, cell-free therapies should be considered a promising alternative for COVID-19 treatment. Clinical trials of EV-based therapies for COVID-19 should clearly describe the dose, route of administration, characteristics of the administered EVs, timing of administration, any monitoring performed during administration, and detailed primary and secondary outcomes.

## Author Contributions

All authors conceived the concept of this article and wrote the manuscript.

## Conflict of Interest

The authors declare that the research was conducted in the absence of any commercial or financial relationships that could be construed as a potential conflict of interest.

## References

[1] Liu S Peng D Qiu H Yang K Fu Z Zou L Mesenchymal stem cells as a potential therapy for COVID-19. Stem Cell Res Ther. (2020) 11:8–11. 10.1186/s13287-020-01678-8 32366290 PMC7197031

[2] Basiri A Pazhouhnia Z Beheshtizadeh N Hoseinpour M Saghazadeh A. Regenerative medicine in COVID-19 treatment : real opportunities and range of promises. Stem Cell Rev Rep. (2020) 20:1–13. 10.1007/s12015-020-09994-5 32564256 PMC7305935

[3] Saleki K Banazadeh M Saghazadeh A Rezaei N. The involvement of the central nervous system in patients with COVID-19. Rev Neurosci. (2020) 31:453–6. 10.1515/revneuro-2020-0026 32463395

[4] Robba C Battaglini D Pelosi P Rocco PRM Robba C Battaglini D. Multiple organ dysfunction in SARS-CoV-2 : MODS-CoV-2. Expert Rev Respir Med. (2020) 1–4. 10.1080/17476348.2020.1778470. [Epub ahead of print]. 32567404 PMC7441756

[5] Rogers CJ Harman RJ Bunnell BA Schreiber MA Xiang C Wang FS. Rationale for the clinical use of adipose-derived mesenchymal stem cells for COVID-19 patients. J Transl Med. (2020) 18:1–19. 10.1186/s12967-020-02380-2 32423449 PMC7232924

[6] Chrzanowski W Kim SY McClements L. Can stem cells beat COVID-19: advancing stem cells and extracellular vesicles toward mainstream medicine for lung injuries associated with SARS-CoV-2 infections. Front Bioeng Biotechnol. (2020) 8:554. 10.3389/fbioe.2020.00554 32574317 PMC7264098

[7] Battaglini D Robba C Ball L Cruz FF Silva PL Pelosi P. Emerging therapies for COVID-19 pneumonia. Expert Opin Investig Drugs. (2020) 1–5. 10.1080/13543784.2020.1771694. [Epub ahead of print]. 32419517 PMC7441765

[8] Russell CD Millar JE Baillie JK. Clinical evidence does not support corticosteroid treatment for 2019-nCoV lung injury. Lancet. (2020) 395:473–5. 10.1016/S0140-6736(20)30317-2 32043983 PMC7134694

[9] RECOVERY Collaborative Group Horby P Lim WS Emberson JR Mafham M Bell JL. Dexamethasone in hospitalized patients with Covid-19 - preliminary report. N Engl J Med. (2020). 10.1056/NEJMoa2021436. [Epub ahead of print]. 32678530 PMC7383595

[10] Golchin A Seyedjafari E Ardeshirylajimi A Mesenchymal stem cell therapy for COVID-19: present or future. Stem Cell Rev Rep. (2020) 16:427–33. 10.1007/s12015-020-09973-w 32281052 PMC7152513

[11] Leng Z Zhu R Hou W Feng Y Yang Y Han Q. Transplantation of ACE2- mesenchymal stem cells improves the outcome of patients with COVID-19 pneumonia. Aging Dis. (2020) 11:216. 10.14336/AD.2020.0228 32257537 PMC7069465

[12] Wang J Jiang M Chen X Montaner LJ. Cytokine storm and leukocyte changes in mild versus severe SARS-CoV-2 infection: review of 3939 COVID-19 patients in China and emerging pathogenesis and therapy concepts. J Leukoc Biol. (2020) 108:17–41. 10.1002/JLB.3COVR0520-272R 32534467 PMC7323250

[13] Ulrich H Pillat MM. CD147 as a target for COVID-19 treatment: suggested effects of azithromycin and stem cell engagement. Stem Cell Rev Rep. (2020) 16:434–40. 10.1007/s12015-020-09976-7 32307653 PMC7167302

[14] Khoury M Cuenca J Cruz FF Figueroa FE Rocco PRM Weiss DJ. Current status of cell-based therapies for respiratory virus infections: applicability to COVID-19. Eur Respir J. (2020) 55:2000858. 10.1183/13993003.00858-2020 32265310 PMC7144273

[15] Cancio M Ciccocioppo R Rocco P Levine B Bronte V Bollard CM. Emerging trends in COVID-19 treatment: learning from inflammatory conditions associated with cellular therapies. Cytotherapy. (2020) 22:474–81. 10.1016/j.jcyt.2020.04.100 32565132 PMC7252029

[16] Mahida RY Matsumoto S Matthay MA. Extracellular vesicles : a new frontier for research in acute respiratory. Am J Respir Cell Mol Biol. (2020) 63:15–24. 10.1165/rcmb.2019-0447TR 32109144 PMC7328246

[17] Bari E Ferrarotti I Saracino L Perteghella S Torre ML Corsico AG. Mesenchymal stromal cell secretome for severe COVID-19 infections: premises for the therapeutic use. Cells. (2020) 9:5–9. 10.3390/cells9040924 32283815 PMC7226831

[18] Lopes-Pacheco M Robba C Rocco PRM Pelosi P. Current understanding of the therapeutic benefits of mesenchymal stem cells in acute respiratory distress syndrome. Cell Biol Toxicol. (2020) 36:83–102. 10.1007/s10565-019-09493-5 31485828 PMC7222160

[19] Borger V Weiss DJ Anderson JD Bussolati B Carter DRF Dominici M. International Society for Extracellular Vesicles and International Society for Cell and Gene Therapy statement on extracellular vesicles from mesenchymal stromal cells and other cells: considerations for potential therapeutic agents to suppress coronavirus disease-19. Cytotherapy. (2020) 22:482–5. 10.1016/j.jcyt.2020.05.002 32425691 PMC7229942

[20] Teixeira FG Panchalingam KM Assunção-Silva R Serra SC Mendes-Pinheiro B Patrício P. Modulation of the mesenchymal Stem cell secretome using computer-controlled bioreactors: impact on neuronal cell proliferation, survival and differentiation. Sci Rep. (2016) 6:27791. 10.1038/srep27791 27301770 PMC4908397

[21] Fatima F Ekstrom K Nazarenko I Maugeri M Valadi H Hill AF. Non-coding RNAs in mesenchymal stem cell-derived extracellular vesicles: deciphering regulatory roles in stem cell potency, inflammatory resolve, and tissue regeneration. Front Genet. (2017) 8:161. 10.3389/fgene.2017.00161 29123544 PMC5662888

[22] Phelps J Sanati-Nezhad A Ungrin M Duncan NA Sen A. Bioprocessing of mesenchymal stem cells and their derivatives: toward cell-free therapeutics. Stem Cells Int. (2018) 2018:9415367. 10.1155/2018/9415367 30275839 PMC6157150

[23] Lim SK Giebel B Weiss DJ Witwer KW Rohde E. Re: Exosomes derived from bone marrow mesenchymal stem cells as treatment for severe COVID-19 by Sengupta et al. Stem Cells Dev. (2020) 29:877–8. 10.1089/scd.2020.0089 32520641 PMC7374615

[24] Maron-Gutierrez T Laffey JG Pelosi P Rocco PRM. Cell-based therapies for the acute respiratory distress syndrome. Curr Opin Crit Care. (2014) 20:122–31. 10.1097/MCC.0000000000000061 24300620

[25] Islam MN Das SR Emin MT Wei M Sun L Westphalen K. Mitochondrial transfer from bone-marrow-derived stromal cells to pulmonary alveoli protects against acute lung injury. Nat Med. (2012) 18:759–65. 10.1038/nm.2736 22504485 PMC3727429

[26] Court AC Le-Gatt A Luz-Crawford P Parra E Aliaga-Tobar V Bátiz LF. Mitochondrial transfer from MSCs to T cells induces treg differentiation and restricts inflammatory response. EMBO Rep. (2020) 21:e48052. 10.15252/embr.201948052 31984629 PMC7001501

[27] Huang C Wang Y Li X Ren L Zhao J Hu Y. Clinical features of patients infected with 2019 novel coronavirus in Wuhan, China. Lancet. (2020) 395:497–506. 10.1016/S0140-6736(20)30183-5 31986264 PMC7159299

[28] Harrell C Fellabaum C Jovicic N Djonov V Arsenijevic N Volarevic V. Molecular mechanisms responsible for therapeutic potential of mesenchymal stem cell-derived secretome. Cells. (2019) 8:467. 10.3390/cells8050467 31100966 PMC6562906

[29] Cheung MB Sampayo-Escobar V Green R Moore ML Mohapatra S Mohapatra SS. Respiratory syncytial virus-infected mesenchymal stem cells regulate immunity via interferon beta and indoleamine-2,3-dioxygenase. PLoS ONE. (2016) 11:e0163709. 10.1371/journal.pone.0163709 27695127 PMC5047639

[30] Picard M McEwen BS Epel ES Sandi C. An energetic view of stress: focus on mitochondria. Front Neuroendocrinol. (2018) 49:72–85. 10.1016/j.yfrne.2018.01.001 29339091 PMC5964020

[31] Manoli I Alesci S Blackman MR Su YA Rennert OM Chrousos GP. Mitochondria as key components of the stress response. Trends Endocrinol Metab. (2007) 18:190–8. 10.1016/j.tem.2007.04.004 17500006

[32] Singer M Deutschman CS Seymour C Shankar-Hari M Annane D Bauer M The third international consensus definitions for sepsis and septic shock (sepsis-3). JAMA. (2016) 315:801–10. 10.1001/jama.2016.0287 26903338 PMC4968574

[33] Heming N Mazeraud A Verdonk F Bozza FA Chrétien F Sharshar T. Neuroanatomy of sepsis-associated encephalopathy. Crit Care. (2017) 21:65. 10.1186/s13054-017-1643-z 28320461 PMC5360026

[34] Phinney DG Di Giuseppe M Njah J Sala E Shiva S St Croix CM. Mesenchymal stem cells use extracellular vesicles to outsource mitophagy and shuttle microRNAs. Nat Commun. (2015) 6:8472. 10.1038/ncomms9472 26442449 PMC4598952

[35] Puhm F Afonyushkin T Resch U Obermayer G Rohde M Penz T. Mitochondria are a subset of extracellular vesicles released by activated monocytes and induce type I IFN and TNF responses in endothelial cells. Circ Res. (2019) 125:43–52. 10.1161/CIRCRESAHA.118.314601 31219742

[36] Sinha P Matthay MA Calfee CS. Is a Cytokine storm relevant to COVID-19? JAMA Intern Med. (2020) 6–8. 10.1001/jamainternmed.2020.3313. [Epub ahead of print]. 32602883

[37] Singer M. The role of mitochondrial dysfunction in sepsis-induced multi-organ failure. Virulence. (2014) 5:66–72. 10.4161/viru.26907 24185508 PMC3916385

[38] Fatima F Nawaz M Nexus between extracellular vesicles, immunomodulation and tissue remodeling: for good or for bad? Ann Transl Med. (2017) 5:139 10.21037/atm.2017.03.71 28462219 PMC5395472

[39] Nawaz M Shah N Zanetti B Maugeri M Silvestre R Fatima F. Extracellular vesicles and matrix remodeling enzymes: the emerging roles in extracellular matrix remodeling, progression of diseases and tissue repair. Cells. (2018) 7:167. 10.3390/cells7100167 30322133 PMC6210724

[40] Théry C Witwer KW Aikawa E Alcaraz MJ Anderson JD Andriantsitohaina R. Minimal information for studies of extracellular vesicles 2018 (MISEV2018): a position statement of the International Society for Extracellular Vesicles and update of the MISEV2014 guidelines. J Extracell Vesicles. (2019) 8:1535750. 10.1080/20013078.2018.1535750 30637094 PMC6322352

[41] Hassanpour M Rezaie J Nouri M Panahi Y. The role of extracellular vesicles in COVID-19 virus infection. Infect Genet Evol. (2020) 85:104422. 10.1016/j.meegid.2020.104422 32544615 PMC7293471

[42] Joo HS Suh JH Lee HJ Bang ES Lee JM. Current knowledge and future perspectives on mesenchymal stem cell-derived exosomes as a new therapeutic agent. Int J Mol Sci. (2020) 21:727. 10.3390/ijms21030727 31979113 PMC7036914

[43] Qiu G Zheng G Ge M Wang J Huang R Shu Q. Functional proteins of mesenchymal stem cell-derived extracellular vesicles. Stem Cell Res Ther. (2019) 10:359. 10.1186/s13287-019-1484-6 31779700 PMC6883709

[44] Maron-Gutierrez T Silva JD Asensi KD Bakker-Abreu I Shan Y Diaz BL. Effects of mesenchymal stem cell therapy on the time course of pulmonary remodeling depend on the etiology of lung injury in mice. Crit Care Med. (2013) 41:319–33. 10.1097/CCM.0b013e31828a663e 23760104

[45] Giebel B Hermann DM. Identification of the right cell sources for the production of therapeutically active extracellular vesicles in ischemic stroke. Ann Transl Med. (2019) 7:188. 10.21037/atm.2019.03.49 31205906 PMC6545303

[46] Zhu YG Feng XM Abbott J Fang XH Hao Q Monsel A. Human mesenchymal stem cell microvesicles for treatment of *Escherichia coli* endotoxin-induced acute lung injury in mice. Stem Cells. (2014) 32:116–25. 10.1002/stem.1504 23939814 PMC3947321

[47] Monsel A Zhu YG Gennai S Hao Q Hu S Rouby JJ. Therapeutic effects of human mesenchymal stem cell-derived microvesicles in severe pneumonia in mice. Am J Respir Crit Care Med. (2015) 192:324–36. 10.1164/rccm.201410-1765OC 26067592 PMC4584251

[48] Khatri M Richardson LA Meulia T. Mesenchymal stem cell-derived extracellular vesicles attenuate influenza virus-induced acute lung injury in a pig model. Stem Cell Res Ther. (2018) 9:17. 10.1186/s13287-018-0774-8 29378639 PMC5789598

[49] Shah TG Predescu D Predescu S. Mesenchymal stem cells-derived extracellular vesicles in acute respiratory distress syndrome: a review of current literature and potential future treatment options. Clin Transl Med. (2019) 8:25. 10.1186/s40169-019-0242-9 31512000 PMC6739436

[50] Wu X Liu Z Hu L Gu W Zhu L. Exosomes derived from endothelial progenitor cells ameliorate acute lung injury by transferring miR-126. Exp Cell Res. (2018) 370:13–23. 10.1016/j.yexcr.2018.06.003 29883714

[51] Abreu SC Weiss DJ Rocco PRM. Extracellular vesicles derived from mesenchymal stromal cells: a therapeutic option in respiratory diseases? Stem Cell Res Ther. (2016) 7:53. 10.1186/s13287-016-0317-0 27075363 PMC4831172

[52] Hao Q Gudapati V Monsel A Park JH Hu S Kato H. Mesenchymal stem cell–derived extracellular vesicles decrease lung injury in mice. J Immunol. (2019) 203:1961–72. 10.4049/jimmunol.1801534 31451675 PMC6760999

[53] Wang C Börger V Sardari M Murke F Skuljec J Pul R. Mesenchymal stromal cell-derived small extracellular vesicles induce ischemic neuroprotection by modulating leukocytes and specifically neutrophils. Stroke. (2020) 51:1825–34. 10.1161/STROKEAHA.119.028012 32312217

[54] Doeppner TR Herz J Görgens A Schlechter J Ludwig A-K Radtke S. Extracellular vesicles improve post-stroke neuroregeneration and prevent postischemic immunosuppression. Stem Cells Transl Med. (2015) 4:1131–43. 10.5966/sctm.2015-0078 26339036 PMC4572905

[55] Sengupta V Sengupta S Lazo A Woods P Nolan A Bremer N Exosomes derived from bone marrow mesenchymal stem cells as treatment for severe COVID-19. Stem Cells Dev. (2020) 29:747–54. 10.1089/scd.2020.0080 32380908 PMC7310206

[56] George MJ Prabhakara K Toledano-Furman NE Wang YW Gill BS Wade CE. Clinical cellular therapeutics accelerate clot formation. Stem Cells Transl Med. (2018) 7:731–9. 10.1002/sctm.18-0015 30070065 PMC6186273

[57] Moll G Rasmusson-Duprez I Von Bahr L Connolly-Andersen AM Elgue G Funke L. Are therapeutic human mesenchymal stromal cells compatible with human blood? Stem Cells. (2012) 30:1565–74. 10.1002/stem.1111 22522999

[58] Chance TC Rathbone CR Kamucheka RM Peltier GC Cap AP Bynum JA. The effects of cell type and culture condition on the procoagulant activity of human mesenchymal stromal cell-derived extracellular vesicles. J Trauma Acute Care Surg. (2019) 87:S74–82. 10.1097/TA.0000000000002225 31246910

[59] Silachev D Goryunov K Shpilyuk M Beznoschenko O Morozova N Kraevaya E. Effect of MSCs and MSC-derived extracellular vesicles on human blood coagulation. Cells. (2019) 8:258. 10.3390/cells8030258 30893822 PMC6468445

[60] Huang R Qin C Wang J Hu Y Zheng G Qiu G. Differential effects of extracellular vesicles from aging and young mesenchymal stem cells in acute lung injury. Aging. (2019) 11:7996–8014. 10.18632/aging.102314 31575829 PMC6781978

[61] Kordelas L Rebmann V Ludwig AK Radtke S Ruesing J Doeppner TR. MSC-derived exosomes: a novel tool to treat therapy-refractory graft-versus-host disease. Leukemia. (2014) 28:970–3. 10.1038/leu.2014.41 24445866

[62] Wang YD Li Y Xu Bin G Dong XY Yang XA Feng ZR. Detection of antibodies against SARS-CoV in serum from SARS-infected donors with ELISA and western blot. Clin Immunol. (2004) 113:145–50. 10.1016/j.clim.2004.07.003 15451470 PMC7106230

[63] Kuate S Cinatl J Doerr HW Überla K. Exosomal vaccines containing the S protein of the SARS coronavirus induce high levels of neutralizing antibodies. Virology. (2007) 362:26–37. 10.1016/j.virol.2006.12.011 17258782 PMC7103344

